# A211 INCIDENCE OF PRIMARY SCLEROSING CHOLANGITIS: A META-ANALYSIS OF POPULATION-BASED STUDIES

**DOI:** 10.1093/jcag/gwab049.210

**Published:** 2022-02-21

**Authors:** J Cooper, A Markovinovic, S Coward, A M Shaheen, M Swain, R Panaccione, C Ma, K L Novak, G G Kaplan

**Affiliations:** Internal Medicine, University of Calgary, Calgary, AB, Canada

## Abstract

**Background:**

Primary sclerosing cholangitis (PSC) is a chronic liver disease associated with significant morbidity, mortality and healthcare utilization. Understanding the incidence of PSC is important in defining the burden of disease and planning for allocation of healthcare resources.

**Aims:**

To conduct a systematic review and meta-analysis of population-based studies of the incidence of PSC and to assess temporal trends of incidence overtime.

**Methods:**

Medline and Embase (from inception to May 10, 2021) were systematically searched to identify studies via the following inclusion criteria: 1) original articles, 2) population-based study of defined geographic area, 3) reported the incidence of PSC or provided data to calculate the incidence of PSC. Studies that assessed specific populations (e.g., pediatric-only, IBD-only) or reported less than 1 year of data were excluded. Abstracts and full texts were reviewed for inclusion and data was extracted independently in duplicate by two individuals (JC, AM). Meta-analyses were performed to calculate overall and country-specific incidence rates (per 100,000 persons) with 95% confidence intervals (CI). Meta-regression calculated the Average Annual Percentage Change (AAPC) of PSC incidence rates overtime.

**Results:**

The initial search returned 3,958 abstracts. After duplicates were removed, abstracts (3,443) were screened, and full texts were reviewed (317), 17 studies met the criteria for inclusion and underwent data extraction. Meta-analysis included 6 studies with annual data contributing to the calculation of AAPC. Studies originated from 10 countries from North America, Europe, and Oceania; however, no population-based studies were published in Asia, Africa, or Latin America (Figure 1). Overall, the incidence rates of PSC was 0.82 per 100,000 (95% CI: 0.62, 1.02) (Figure 1). Incidence rates of PSC were significantly increasing overtime (AAPC: 4.56%; 95% CI: 0.45, 8.68).

**Conclusions:**

The incidence of PSC is low at 0.82 per 100,000 but has been significantly increasing over time. Future studies on the incidence of PSC should be directed at Asia, Africa of Latin America to assess the global epidemiology of PSC.

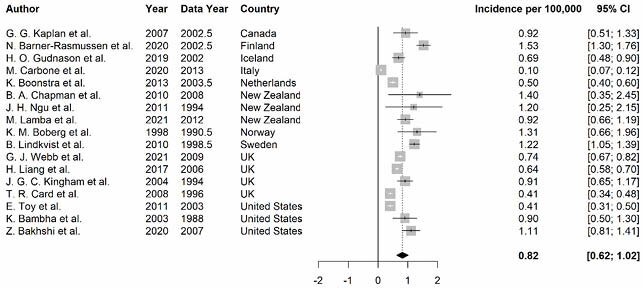

Figure 1: Pooled incidence rate estimates of PSC per 100,000 person-years at risk.

**Funding Agencies:**

None

